# The safety of pericardiocentesis in patients under antithrombotic therapy: A single-center experience

**DOI:** 10.3389/fcvm.2022.1013979

**Published:** 2022-09-21

**Authors:** Yuansong Zhu, Chengxiang Zhang, Yuqiao Xie, Bryan Richard Sasmita, Zhenxian Xiang, Yi Jiang, Ming Gong, Yaxin Wang, Siyu Chen, Suxin Luo, Bi Huang

**Affiliations:** ^1^Department of Cardiology, The First Affiliated Hospital of Chongqing Medical University, Chongqing, China; ^2^The First Clinical College, Chongqing Medical University, Chongqing, China

**Keywords:** pericardiocentesis, antiplatelet, anticoagulation, bleeding, pericardial effusion

## Abstract

**Objective:**

This study aimed to analyze the characteristics of patients with pericardial effusion requiring pericardiocentesis and to evaluate the safety of pericardiocentesis without discontinuation of anticoagulant or antiplatelet drugs.

**Methods:**

We performed a retrospective study of patients undergoing pericardiocentesis in our hospital between 2012 and 2022. Patients were categorized into the Antithrombotic Group if they had used any antiplatelet or anticoagulant drugs on the day of pericardiocentesis; otherwise they were categorized into the Non-antithrombotic Group. All procedures were performed by experienced cardiologists with echocardiographic guidance. Bleeding events were defined using the National Institutes of Health scale of adverse events.

**Results:**

A total of 501 consecutive patients were identified and 70 cases were under antithrombotic drugs (Antithrombotic Group). Patients in Antithrombotic Group were older, had more comorbidities, presented with lower platelet counts and prolonged activated partial thromboplastin time (all *p* < 0.05). Malignancy was the most common etiology for pericardial effusion in both groups (28.6% in Antithrombotic Group and 54.7% in Non-antithrombotic Group) and tuberculosis was the second etiology in the Non-antithrombotic Group (21.9%), while procedure-related effusion (17.1%) accounted for the second cause in the Antithrombotic Group. Two patients in the Antithrombotic Group had mild oozing at the puncture site that resolved without interventions (2.9 vs. 0%, *p* = 0.019), and no bleeding events higher than Grade 1 occurred in either group.

**Conclusion:**

Although antiplatelet or anticoagulant drugs may put patients undergoing pericardiocentesis at theoretically higher risk of bleeding, our study demonstrated that they are not associated with increased major bleeding complications.

## Introduction

Pericardiocentesis is an important procedure for either diagnostic or therapeutic purposes and is often performed in an emergency setting (1–4). It is a procedure with a certain rate of complications, including hemothorax, pneumothorax, pneumopericardium, arrhythmias, and coronary artery or cardiac chamber puncture (5). The 2005 European Society of Cardiology guideline of pericardial diseases suggested aortic dissection as a major contraindication for pericardiocentesis and uncorrected coagulopathy, anticoagulant therapy, and thrombocytopenia with < 50^*^10^9^/L as relative contraindications (6).

Ideally, the contraindications should be modified to minimize risks for diagnostic procedures for pericardial effusion. However, as many patients with pericardial effusion have comorbidities and are under the treatment of antiplatelet or anticoagulant drugs, physicians often encounter doubts about whether pericardiocentesis is safe without discontinuing these drugs, since periprocedural discontinuation of these drugs may put patients with cardiovascular or cerebrovascular diseases at increased risk of thrombosis (7, 8). Moreover, in patients with cardiac tamponade that causes hemodynamic instability, urgent pericardiocentesis could be the only life-saving treatment. In the present study, we aimed to analyze the characteristics of patients with pericardial effusion requiring pericardiocentesis and to evaluate the safety of pericardiocentesis without discontinuation of antiplatelet or anticoagulant drugs.

## Methods

Consecutive patients with pericardial effusion requiring pericardiocentesis admitted to The First Affiliated Hospital of Chongqing Medical University between January 2012 and May 2022 were included. All procedures were performed by experienced cardiologists under echocardiographic guidance. The study was approved by the medical ethics committee of our hospital, and the need for informed consent was waived due to the observational design.

The sample size of the study was calculated using a simple formula for pilot studies that has been widely cited previously (9). The study by Ryu et al. (10) that aimed to investigate bleeding complications of pericardiocentesis has detected a bleeding rate of 0.56% in a consecutive cohort. We adopted 0.56% as the probability and 95% as confidence level and finally determined a sample size of 533 patients. We later searched all patients undergoing pericardiocentesis in our hospital medical record system and identified 501 patients. Patients were categorized into the Antithrombotic Group if they had used any of the following drugs on the day of the pericardiocentesis: aspirin, clopidogrel, ticagrelor, warfarin, nonvitamin K antagonist oral anticoagulants (NOAC), unfractionated heparin, and low molecular weight heparin. Otherwise patients were categorized into the Non-antithrombotic Group. The baseline characteristics, medications, laboratory results, and procedural details of all patients were collected through the electronic medical records. Laboratory results of hemoglobin, hematocrit, platelet count, international normalized ratio (INR), prothrombin activity (PTA), activated partial thromboplastin time (APTT) were acquired in all patients. Serum albumin (3.6%) and creatinine (3.2%) were missing in a small proportion of patients and substituted by the median value of their group. Pericardiocentesis was deemed diagnostic if the etiology of pericardial effusion was unclear at admission and therapeutic if the procedure aimed to relieve patients' symptoms. The procedure could be both diagnostic and therapeutic. Other procedural details of the entry points, the color of pericardial effusion, whether drainage was performed, and the volume of drainage were also recorded.

The etiologies of pericardial effusion were judged using the following criteria. The effusion was deemed as malignant when pericardial fluid cytology detected atypical or overtly malignant cells. Patients with known previous malignancy but negative cytology results were also classified into this group. If malignancy was diagnosed for the first time, patients would undergo imaging tests or biopsies to determine the primary lesion. Procedure-related effusion indicated newly appeared pericardial effusion after invasive cardiac procedures. Tuberculosis was diagnosed by positive results of mycobacteria smear or culture of the effusion. When mycobacteria could not be detected, patients were also classified into this group if tuberculosis was highly suspected according to the effusion routine and biochemistry tests and anti-tuberculosis drugs were given. Autoimmune disease referred to effusion related to autoimmune diseases such as systemic lupus erythematosus. Acute coronary syndrome was diagnosed based on dynamic electrocardiograms and cardiac enzymes. Effusion due to heart failure was diagnosed based on clinical symptoms, left ventricular ejection fraction, brain natriuretic peptide and excluding other causes. Hypothyroidism was diagnosed based on elevated thyroid-stimulating hormone and decreased triiodothyronine and thyroxine. Uremia-related effusion was diagnosed when dialysis dependency existed in the absence of other possible explanations. Infection was confirmed by effusion routine and biochemistry tests, venous inflammatory indexes, and blood or effusion culture. Aortic dissection was confirmed by echocardiography or computed tomography. The final idiopathic group included patients with no clear explanation using routine clinical care.

Hospitalization days and adverse events of bleeding events, pneumothorax, coronary artery or cardiac chamber puncture and in-hospital mortality were collected from hospital electronic database. Bleeding events were graded using the National Institutes of Health Common Terminology Criteria for Adverse Events version 5.0 (11). A Grade 1 event refers to asymptomatic mild bleeding that does not require intervention; Grade 2 refers to a hemorrhagic complication requiring aspiration or evaluation; Grade 3 refers to a complication requiring transfusion or elective operation; Grade 4 refers to a life-threatening complication requiring urgent intervention; and Grade 5 is hemorrhage leading to death. Bleeding complications were further specified as asymptomatic oozing at the puncture size (Grade 1), symptomatic chest wall hematoma requiring aspiration (Grade 2), a drop of hemoglobin (Hb) > 20 g/L in 48 h (Grade 3), bleeding requiring transfusion (Grade 3), hemothorax and hemopericardium requiring urgent surgery or intervention (Grade 4). Post-procedure Hb, chest imaging, echocardiography and progress notes were evaluated for grading. In our hospital, all patients undergoing pericardiocentesis receive at least 2-h cardiac monitoring after the procedure and physicians are also required to write medical notes 2 h after the procedure and every day thereafter. Thus, the medical records are valid enough to interpret outcomes.

Continuous variables are presented as medians with interquartile ranges (IQRs), and comparisons between groups were achieved using the Mann-Whitney test due to the non-normal distribution and heterogeneity of variance. Categorical variables are presented as percentages and compared using chi-square test, or Fisher's exact test if the expected count was < 5. All statistical analyses were carried out using the SPSS software, version 26.0 (IBM Corp. Armonk, NY, USA). Statistical significance was defined as a two-sided *p* < 0.05.

## Results

### Baseline characteristics and laboratory results of the patients

Among the 501 patients included, 70 patients (14.0%) were under the treatment of antiplatelet or anticoagulant drugs. As presented in Table 1, they were older (Antithrombotic vs. Non-antithrombotic, 66 vs. 59 years, *p* = 0.001) and had more comorbidities of hypertension (47.1 vs. 22.5%, *p* < 0.001), diabetes (25.7 vs. 10.0%, *p* < 0.001), chronic kidney disease (22.9 vs. 7.0%, *p* < 0.001), coronary artery disease (28.6 vs. 9.7%, *p* < 0.001), and atrial fibrillation (18.6 vs. 4.6%, *p* < 0.001). Patients under antithrombotic drugs presented with lower systolic blood pressure (SBP, 115 vs. 120 mmHg, *p* = 0.009) and diastolic blood pressure (DBP, 71 vs. 80 mmHg, *p* < 0.001). They had lower platelet count (210^*^10^9^/L vs. 233^*^10^9^/L, *p* = 0.027) and longer activated partial thromboplastin time (APTT, 30.0 vs. 28.6 s, *p* = 0.005), but the international normalized ratio (INR, 1.15 vs. 1.16, *p* = 0.649) was comparable between the two groups.

**Table 1:** Baseline characteristics and laboratory results of the patients.

	**Total (*n* = 501)**	**Antithrombotic (*n* = 70)**	**Non-antithrombotic (*n* = 431)**	***P***
Age, years	60 (50, 68)	66 (57, 73)	59 (49, 68)	0.001
Male, %	295 (58.9)	46 (65.7)	249 (57.8)	0.210
Hypertension, %	130 (25.9)	33 (47.1)	97 (22.5)	< 0.001
Diabetes, %	61 (12.2)	18 (25.7)	43 (10.0)	< 0.001
Chronic kidney disease, %	46 (9.2)	16 (22.9)	30 (7.0)	< 0.001
Coronary artery disease, %	59 (11.8)	20 (28.6)	42 (9.7)	< 0.001
Atrial fibrillation, %	33 (6.6)	13 (18.6)	20 (4.6)	< 0.001
SBP, mmHg	120 (108, 134)	115 (99, 130)	120 (109, 134)	0.009
DBP, mmHg	78 (70, 89)	71 (64, 78)	80 (70, 90)	< 0.001
HR, beat per minute	102 (86, 115)	98 (83, 116)	102 (86, 114)	0.464
Hemoglobin, g/L	122 (109, 133)	124 (106, 135)	121 (109, 133)	0.933
Hematocrit, %	36.9 (33.2, 40.7)	37.3 (31.8, 41)	36.9 (33.4, 40.5)	0.978
Platelet count, *10^9^/L	229 (170, 306)	210 (133, 276)	233 (175, 311)	0.027
INR	1.16 (1.07, 1.25)	1.15 (1.04, 1.25)	1.16 (1.07, 1.25)	0.649
PTA, %	77.4 (66.2, 90.3)	76.1 (66, 95.6)	77.5 (66.2, 90)	0.963
APTT, s	28.8 (26, 32.7)	30 (27.4, 35.5)	28.6 (25.9, 32.4)	0.005
Albumin, g/L	36 (32, 39)	37 (32, 40)	36 (32, 39)	0.514
Creatinine, μmoI/L	71.5 (60, 92)	90.5 (73, 126)	70 (58, 89)	< 0.001

SBP, systolic blood pressure; DBP, diastolic blood pressure; HR, heart rate; INR, international normalized ratio; PTA, prothrombin activity; APTT, activated partial thromboplastin time.

### Pericardiocentesis-related information of the patients

The pericardiocentesis-related information is showed in Table 2. Pericardiocentesis was therapeutic in almost all patients in both groups (100 vs. 99.8%, *p* = 1.000). In most patients, the procedure also had a diagnostic purpose, and this percentage was lower in the Antithrombotic Group (72.9 vs. 90.7%, *p* < 0.001). The entry ports of pericardiocentesis did not differ between groups. In our center, more than 90% of the pericardiocentesis was performed through subxiphoidal access (94.3 vs. 91.6%, *p* = 0.450). Dark red was the most common color of pericardial effusion (*p* = 0.360). The Non-antithrombotic Group had a higher total drainage volume (600 vs. 800 mL, *p* = 0.045).

**Table 2:** Pericardiocentesis-related information of the patients.

	**Total (*n* = 501)**	**Antithrombotic (*n* = 70)**	**Non-antithrombotic (*n* = 431)**	***P***
Diagnostic, %	442 (88.2)	51 (72.9)	391 (90.7)	< 0.001
Therapeutic, %	500 (99.8)	70 (100)	430 (99.8)	1.000
Entry ports, %				0.450
Subxiphoidal	461 (92.0)	66 (94.3)	395 (91.6)	
Apical	40 (8.0)	4 (5.7)	36 (8.4)	
Color, %				0.360
Dark red	334 (67.1)	49 (72.1)	285 (66.3)	
Light red	43 (8.6)	5 (7.4)	38 (8.8)	
Dark yellow	9 (1.8)	0 (0)	9 (2.1)	
Light yellow	112 (22.5)	14 (20.6)	98 (22.8)	
Drainage, %	486 (97.0)	65 (92.9)	421 (97.7)	0.069
Volume of drainage, mL	750 (490, 1,160)	600 (210, 1,090)	800 (515, 1,160)	0.045

### Etiologies of the pericardial effusion

The etiologies of the pericardial effusion in all patients are presented in Table 3. Malignancy represented the most common etiology of pericardial effusion in both groups (51.0% in total, 28.6% in Antithrombotic Group, and 54.7% in Non-antithrombotic Group), and lung cancer was the most common cancer (38.9, 22.9, and 41.5%). In the Non-antithrombotic Group, tuberculosis was the second etiology (21.9%), followed by idiopathic pericardial effusion (11.2%). Pericardial effusion caused by cardiac invasive procedures accounted for only 2.1% in this group. In the Antithrombotic Group, pericardial effusion due to cardiac invasive procedures was the second etiology (17.1%), followed by acute coronary syndrome (12.9%) and idiopathic pericardial effusion (12.9%). The incidence of pericardial effusion after radiofrequency ablation (11.4%) was 1-fold higher than effusion after coronary intervention (5.7%).

**Table 3:** Etiologies of the patients undergoing pericardiocentesis.

	**Total (*n* = 501)**	**Antithrombotic (*n* = 70)**	**Non-antithrombotic (*n* = 431)**
Malignancy	255 (51.0)	20 (28.6)	235 (54.7)
Lung cancer	195 (38.9)	16 (22.9)	179 (41.5)
Breast cancer	11 (2.2)	1 (1.4)	10 (2.3)
Mediastinum tumor	9 (1.8)	0 (0)	9 (2.1)
Hematological malignancy	3 (0.6)	0 (0)	3 (0.7)
Endometrial carcinoma	3 (0.6)	0 (0)	3 (0.7)
Cardiac tumor	2 (0.4)	0 (0)	2 (0.5)
Gastric cancer	2 (0.4)	0 (0)	2 (0.5)
Prostate cancer	1 (0.2)	0 (0)	1 (0.2)
Liver cancer	1 (0.2)	1 (1.4)	0 (0)
Ovarian cancer	1 (0.2)	0 (0)	1 (0.2)
Colon cancer	1 (0.2)	0 (0)	1 (0.2)
Teratoma	1 (0.2)	0 (0)	1 (0.2)
Nasopharyngeal carcinoma	2 (0.4)	0 (0)	2 (0.5)
Undetermined	23 (4.6)	2 (2.9)	21 (4.9)
Procedure-related	21 (4.2)	12 (17.1)	9 (2.1)
Coronary	4 (0.8)	4 (5.7)	0 (0)
Radiofrequency ablation	15 (3.0)	8 (11.4)	7 (1.6)
LAAO	1 (0.2)	0 (0)	1 (0.2)
Tuberculosis	98 (19.6)	4 (5.7)	94 (21.9)
Autoimmune disease	12 (2.4)	2 (2.9)	10 (2.3)
Acute coronary syndrome	11 (2.2)	9 (12.9)	2 (0.5)
Heart failure	9 (1.8)	5 (7.1)	4 (0.9)
Hypothyroidism	10 (2.0)	1 (1.4)	9 (2.1)
Uremia	11 (2.2)	2 (2.9)	9 (2.1)
Infection	15 (3.0)	4 (5.7)	11 (2.6)
Aortic dissection	2 (0.4)	2 (2.9)	0 (0)
Drug-related	1 (0.2)	0 (0)	1 (0.2)
Idiopathic	57 (11.4)	9 (12.9)	48 (11.2)

LAAO, left atrial appendage occlusion.

### Hospitalization days and adverse events after pericardiocentesis

The outcomes after pericardiocentesis are listed in Table 4. Two patients in the Antithrombotic Group had mild oozing at the puncture site that resolved after compression and did not require other interventions (Antithrombotic vs. Non-antithrombotic, 2.9 vs. 0%, *p* = 0.019). No patients experienced any bleeding events higher than Grade 1. The incidence of pneumothorax after the pericardiocentesis did not differ between the two groups (0 vs. 0.4%, *p* = 1.000). No coronary artery or cardiac chamber puncture events occurred in either group. The Antithrombotic Group has a longer median hospitalization stay than the Non-antithrombotic Group (12.5 vs. 8 days, *p* < 0.001). A total of 22 patients (4.4%) undergoing pericardiocentesis died during hospitalization. The in-hospital mortality rate was higher in the Antithrombotic Group than in the Non-antithrombotic Group (11.4 vs. 3.2%, *p* = 0.005). A subgroup analysis of complications of pericardiocentesis in tuberculosis was performed, as showed in Table 5. There was a total of 98 procedures due to tuberculosis and no adverse events occurred. One patient (1.0%) died during hospitalization and the mortality rate was lower than in the total population (4.4%).

**Table 4:** Adverse events and hospitalization days of patients undergoing pericardiocentesis.

	**Total (*n* = 501)**	**Antithrombotic (*n* = 70)**	**Non-antithrombotic (*n* = 431)**	***P***
Bleedings, any	2 (0.4)	2 (2.9)	0 (0)	0.019
Bleedings, Grade 1	2 (0.4)	2 (2.9)	0 (0)	0.019
Bleedings, Grade 2–5	0 (0)	0 (0)	0 (0)	1.000
Pneumothorax	2 (0.5)	0 (0)	2 (0.4)	1.000
Coronary artery or cardiac chamber puncture	0 (0)	0 (0)	0 (0)	1.000
Hospitalization, days	9 (5.5, 13)	12.5 (8, 19)	8 (5, 12)	< 0.001
In-hospital mortality	22 (4.4)	8 (11.4)	14 (3.2)	0.005

**Table 5:** Characteristics of patients of tuberculosis-related pericardial effusions.

	**Tuberculosis (*n* = 98)**
Bleedings, any	0 (0)
Bleedings, Grade 1	0 (0)
Bleedings, Grade 2–5	0 (0)
Pneumothorax	0 (0)
Coronary artery or cardiac chamber puncture	0 (0)
Hospitalization, days	10 (7, 13)
In-hospital mortality	1 (1.0%)

### Analysis of different antithrombotic schemes

**Figure 1:**
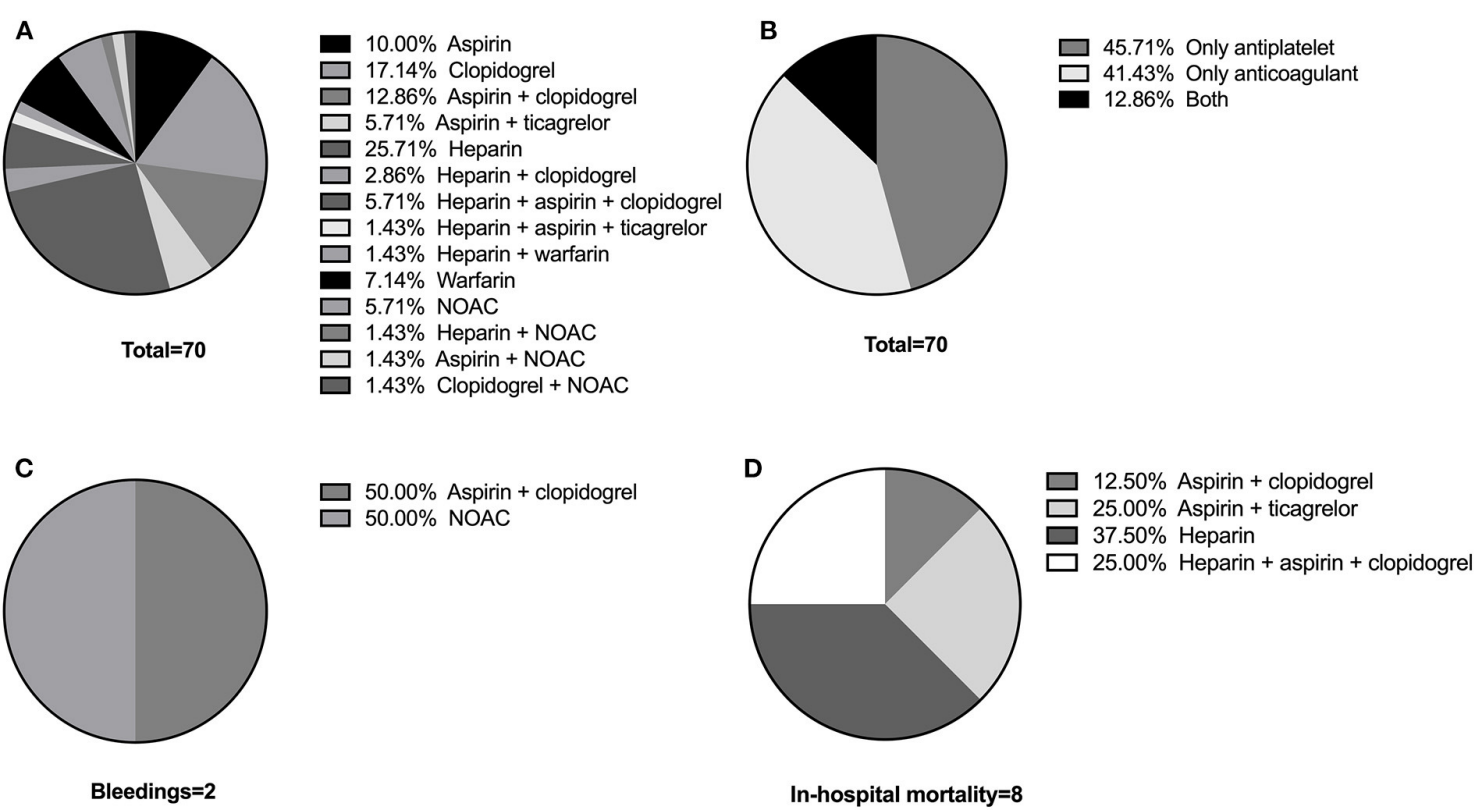
**(A)** The exact antithrombotic schemes of the Antithrombotic Group. **(B)** Proportion of patients under antiplatelet drugs, anticoagulant drugs, and both. **(C)** Proportion of patients that had Grade 1 bleeding events on different schemes. **(D)** Proportion of patients that died during hospitalization on different schemes.

Table 6 lists the exact number of patients under different antithrombotic schemes and their respective adverse events. The percentages are also illustrated in Figure 1. The most common scheme is heparin (25.71%), followed by clopidogrel (17.14%), aspirin + clopidogrel (12.86%), and aspirin (10%) (Figure 1A). Among the 70 patients in Antithrombotic Group, 45.71% were under antiplatelet drugs and 41.43% were under anticoagulants, while 12.86% received both (Figure 1B). Of the 2 patients that had Grade 1 bleeding events, 1 patient was on aspirin + clopidogrel and 1 was on NOAC (Figure 1C). Among the 8 patients under antithrombotic therapy that died during hospitalization, 1 patient (12.5%) was on aspirin + clopidogrel, 2 (25.0%) on aspirin + ticagrelor, 3 (37.5%) on heparin and 2 (25.0%) on heparin + aspirin + clopidogrel (Figure 1D).

**Table 6:** The number of patients under different antithrombotic schemes and respective adverse events.

	**Patients**	**Bleedings**	**Pneumothorax**	**In-hospital mortality**
Non-antithrombotic	431 (86.0)	0	2	14
Aspirin	7 (1.4)	0	0	0
Clopidogrel	12 (2.4)	0	0	0
Aspirin + clopidogrel	9 (1.8)	1	0	1
Aspirin + ticagrelor	4 (0.8)	0	0	2
Heparin	18 (3.6)	0	0	3
Heparin + clopidogrel	2 (0.4)	0	0	0
Heparin + aspirin + clopidogrel	4 (0.8)	0	0	2
Heparin + aspirin + ticagrelor	1 (0.2)	0	0	0
Heparin + warfarin	1 (0.2)	0	0	0
Warfarin	5 (1.0)	0	0	0
NOAC	4 (0.8)	1	0	0
Heparin + NOAC	1 (0.2)	0	0	0
Aspirin + NOAC	1 (0.2)	0	0	0
Clopidogrel + NOAC	1 (0.2)	0	0	0

NOAC, non-vitamin K antagonist oral anticoagulants.

## Discussion

This single-center, retrospective analysis provided baseline characteristics and complication data in patients undergoing pericardiocentesis without withdrawing antiplatelet or anticoagulant drugs. Our study demonstrated that although patients without withdrawing antiplatelet or anticoagulant drugs were at theoretically higher risk of bleeding, major bleeding complications of pericardiocentesis were uncommon. Pericardiocentesis in patients under anticoagulants or antiplatelet drugs may be performed safely without increasing major bleeding complications.

Pericardiocentesis is one of the most common puncture procedures and can be a life-saving treatment especially for patients with cardiac tamponade. However, the procedure may be associated with injuries of adjacent blood vessels or important organs such as lung, liver, coronary artery and even cardiac chamber, and thus results in increased risk of bleeding events, especially in patients with coagulation disorders or concomitant usage of antithrombotic drugs. Whether it is safe to perform pericardiocentesis in these patients is a realistic problem facing the cardiologists during clinical practice. In fact, whether punctures are safe under antithrombotic therapy in the thoracic or abdominal cavity has been investigated previously. For pleural procedures including thoracentesis and small-bore chest tube placement, studies have demonstrated that the usage of antiplatelet drugs is not associated with excessive bleeding events (12–15). Patel et al. (16) later extended the conclusion to patients under NOAC. For abdominal paracentesis, a study that included 32 Budd-Chiari Syndrome patients under oral anticoagulation while requiring abdominal paracentesis at the same time also found no bleeding events (17). However, there are not enough clinical data regarding the safety of pericardiocentesis in patients under antiplatelet or anticoagulant drugs. Several cases have reported its feasibility in patients with uncorrected coagulopathy, such as thrombocytopenia or elevated INR due to cancer, chemotherapy, and cirrhosis (18–20). Iliescu et al. (21) analyzed cancer patients presenting with cardiac tamponade in the setting of thrombocytopenia and identified 2 major bleeding events out of 60 procedures. Ryu et al. (10) recently performed a retrospective analysis of a large cohort of 1,048 echocardiographic-guided pericardiocentesis. It was demonstrated that the overall significant bleeding rate was low at 0.57% and that neither the presence of coagulopathy nor thrombocytopenia was associated with significant bleeding events, suggesting that pericardiocentesis might also be safe in patients with antithrombotic therapy.

The present study found that patients under antithrombotic drugs were older, presented with lower platelet count and abnormal coagulation parameters, all of which are actual factors that physicians hesitate to perform pericardiocentesis. Theoretically, these risk factors could indeed expose patients to an increased risk of bleeding; however, our study found no major bleeding events in these patients. We believe that the guidance of echocardiography might take most of the credit, since the echocardiography could clearly show the important structures adjacent to the pericardium and reduce the possibility of injuries of big blood vessels and organs. With these big vessels and organed being avoided, even if the procedure causes injuries of small vessels that could not be detected by echocardiography, they are less likely to lead to major bleeding events.

Another finding of the present study is that patients who were under antithrombotic drugs and without antithrombotic drugs showed significantly different etiologies of the pericardial effusion. Since patients under antithrombotic drugs had different comorbidities, there is significant variation in the exact antithrombotic schemes among these patients. Most patients received only antiplatelet drugs or anticoagulants, but a small proportion of patients received both. In the present study, two cases of Grade 1 bleeding events occurred, of which one was on aspirin + clopidogrel and the other on NOAC. Whether the bleeding events were associated with exact antithrombotic drugs remained to be clarified. However, among the wide antithrombotic schemes, the incidence of bleeding was rare, indicating the relative safety of pericardiocentesis under antithrombotic drugs. Although malignancy represents the most common cause of the effusion of both groups, similar to the data reported by some previous studies in the recent decade (22–24), its percentage in the Antithrombotic Group (28.6%) was much lower than the Non-antithrombotic Group (54.7%). Procedure-related effusion accounted for the second etiology for the Antithrombotic Group (17.1%) and was higher than the Non-antithrombotic Group (2.1%). There are some possible interpretations. First, patients with antithrombotic drugs have more cardiovascular comorbidities, such as hypertension, coronary artery disease and atrial fibrillation; thus, they are more likely to undergo cardiac invasive procedures. Second, antithrombotic drugs may expose patients to higher bleeding risk when undergoing cardiac invasive procedures (25).

The incidence of tuberculosis-related pericardial effusion remained high at 19.6% of the total population in our study, which was significantly increased compared to previous studies based in developed countries (22, 26, 27), but slightly lower than a Chinese study reported 10 years ago (28.6%) (28). As China still ranked second place after India in the total cases of tuberculosis in the world, the disease burden of tuberculosis in China remains high (29, 30). Since the sensitivity of mycobacteria smear or culture is low, the etiology of effusion was also attributed to tuberculosis in our study if it was highly suspected according to the chemical examinations of effusion and diagnostic antituberculosis treatment were given.

The present study has the following clinical implications. The procedure of pericardiocentesis can be safely performed in patients with comorbidities that should receive antiplatelet or anticoagulant treatment, especially under the guidance of echocardiography. The usage of antithrombotic drugs should not be viewed as a contraindication, and the risks of thrombosis of discontinuing antiplatelet or anticoagulant drugs may be avoided. The subgroup analysis showed that the procedure of pericardiocentesis in tuberculosis-related pericardial effusion was safe. Thus, the results are also meaningful for physicians in the developing world where tuberculosis remains the leading cause of pericardial disease (31).

There are several limitations in our study. First, the single-center and retrospective design of the study has limited the applicability of the conclusions to other populations. The real sample size of the study was also slightly smaller than ideal and larger studies are needed to further validate our experience. Second, since the study included patients who finally underwent pericardiocentesis, some patients who have been taking antithrombotic drugs may be deemed at high risk of bleeding at first and pericardiocentesis was not performed in these patients at all. Therefore, there is potential selection bias. Third, the duration and dose range of antiplatelets and anticoagulants use in the Antithrombotic Group were unavailable due to the retrospective design and more studies with prospective design are warranted. Finally, there was a lack of follow-up data in our study. Although the adverse events of pericardiocentesis are most likely to occur within days, a longer follow-up would be helpful to detect late adverse events and to understand their impact on patient prognosis.

## Conclusion

Although antiplatelet or anticoagulant drugs may put patients undergoing pericardiocentesis at theoretically higher risk of bleeding, our study demonstrated that they are not associated with increased major bleeding complications undergoing pericardiocentesis.

## Data availability statement

The datasets presented in this article are not readily available because the data that support the findings of this study are available from the corresponding authors upon reasonable request. Requests to access the datasets should be directed to huangbi120@163.com.

## Ethics statement

The studies involving human participants were reviewed and approved by the First Affiliated Hospital of Chongqing Medical University. Written informed consent for participation was not required for this study in accordance with the national legislation and the institutional requirements. There are no potentially identifiable images or data included in this article.

## Author contributions

YZ and CZ designed the study, participated in the data collection, analyzed the data, and drafted the manuscript. YX, BS, ZX, YJ, MG, YW, and SC participated in the data collection. BH coordinated the process and revised the manuscript. SL provided funding and supervised. All authors take responsibility for all aspects of the reliability of the data presented and their discussed interpretation.

## Conflict of interest

The authors declare that the research was conducted in the absence of any commercial or financial relationships that could be construed as a potential conflict of interest.

## Publisher's note

All claims expressed in this article are solely those of the authors and do not necessarily represent those of their affiliated organizations, or those of the publisher, the editors and the reviewers. Any product that may be evaluated in this article, or claim that may be made by its manufacturer, is not guaranteed or endorsed by the publisher.
